# Relationship dissatisfaction and other risk factors for future relationship dissolution: a population-based study of 18,523 couples

**DOI:** 10.1007/s00127-013-0681-3

**Published:** 2013-03-28

**Authors:** Gun-Mette B. Røsand, Kari Slinning, Espen Røysamb, Kristian Tambs

**Affiliations:** 1 Division of Mental Health, Norwegian Institute of Public Health, PO Box 4404, Nydalen, 0403 Oslo, Norway; 2 National Network for Infant Mental Health, Centre for Child and Adolescent Mental Health Eastern and Southern Norway (R.BUP Oslo), Oslo, Norway; 3 Department of Psychology, University of Oslo, Oslo, Norway

**Keywords:** Relationship dissolution, Relationship satisfaction, Emotional distress, Parents with small children

## Abstract

**Purpose:**

There has been a marked increase in divorce rates in most Western societies over the last 50 years. Relationship dissolution is associated with negative consequences both for adults and children, so it is important to understand the factors that help retain marital stability. The first aim of this prospective study was to identify risk factors for relationship dissolution in 18,523 couples in Norway, with a particular focus on individual dissatisfaction with the relationship. The second aim was to assess interaction effects between relationship dissatisfaction and other predictors of relationship dissolution.

**Methods:**

Pregnant women and their partners enrolled in the Norwegian Mother and Child Cohort study completed questionnaires during the pregnancy that asked about relationship dissatisfaction, strain, demographics, and other risk factors. The main outcome variable was relationship dissolution in the 39-month period from gestational week 30–36 months postpartum. Associations between the risk factors and relationship dissolution were estimated by logistic regression analysis.

**Results:**

Except for younger female age, relationship dissatisfaction in women and lower education in men, were the strongest predictors of relationship dissolution. Another strong factor was women’s persistent strain. No significant interaction effects were found between relationship dissatisfaction and the other variables in the analyses.

**Conclusions:**

Dissatisfaction with the relationship, in particular in women, and low male education are important predictors of relationship dissolution, although other factors are also related to dissolution. There are only few studies on relationship predictors of dissolution conducted in Europe, and the current study adds to this body of knowledge.

## Introduction

Romantic relationships are generally less stable than they used to be. In most Western societies, there has been a large increase in divorce over the last 50 years that peaked in the 1980s [25]. Data from the last 10 years reveal fluctuations in the divorce rates in the United States and in Europe. Irrespective of fluctuations and varying trends in different countries during the last decade, almost all Western countries had a higher rate of divorce in 2007 compared to that in the early 1970s. Divorce rates remain high, and there are few signs of a trend reversal.

The US has the highest divorce rate of any Western nation today [4], with a divorce rate of 3.5 per 1,000 people in 2009, according to the National Centre for Health Statistics. Approximately, one half of all first marriages end in separation or divorce in the US [12, 58], with even higher rates of divorce for second marriages [19]. Although Belgium currently has one of the highest divorce rates in Europe, with 3.3 divorces per 1,000 people in 2008, the level remains substantially lower than in the US. Norway, where the current study was conducted, has the lowest divorce rate in Scandinavia, i.e. 2.1 per 1,000 people in 2008 (Eurostat, 2010). In Norway in 2010, there were 23,600 new marriages, and 10,300 marriages ended in divorce the same year (Statistics Norway, 2011). Cohabiting unions are even more likely to dissolve than are marriages [41, 65]. In Norway, the dissolution risk is three times higher among cohabiters than among married couples, even among couples who have children together [72]. One should bear in mind that there are cultural differences in the attitudes towards cohabitation in different countries. For instance, in the Scandinavian countries, cohabitation is common and more widely accepted than in the US [74] and established as a childbearing institution across the social spectrum rather than being confined to the socially disadvantaged [41]. Fully valid and detailed data on cohabitation and cohabiting breakups are still not available in Norway. An Norwegian study based on the data from 1999 concluded that the separation statistics only identified two-thirds of all children experiencing parental breakup that year [17]. The rest of these children had cohabiting parents who broke their relationship. In 2009, 13,408 children (age 1–17) experienced parental marital separation. If this is two-thirds of all children experiencing parental breakup, this year slightly more than 20,000 (1.8 %) of all Norwegian children was affected by the dissolution of their parents’ relationship (Statistics Norway, 2011).

The health-enhancing properties of personal relationships have been documented repeatedly [35]. On an average, married people enjoy better mental and physical health than do unmarried people, and marriage’s protective effects are notably stronger for men than women [44]. Most studies report that gaining a spouse improves mental health, while loss of a spouse negatively affects mental health [42, 66, 77]. Previous studies have also documented links between divorce and mental and physical health. When compared with married individuals, divorced and separated individuals tend to have poorer mental and physical health than continuously married individuals both in the US [77, 79] and in European countries, including Norway, Sweden, and England [28, 49, 53]. In addition to emotional and physical health consequences, there are also major social and financial implications for divorcing and separating couples [1]. Further, the characteristics of the marital relationship might influence the effects of divorce on the mental health of the partners. For example, some research indicates that people in low-quality marriages benefit from divorce [3]. In addition to the possible consequences for the adult partners involved, a large number of epidemiological studies conclude with small, but significant differences in the adjustment and well being of children of divorced parents as compared to children with no experience of divorce [2, 5, 38]. Despite the small mean effects, the high prevalence of divorce leads to a substantial number of children with various adjustment problems. Thus, divorce and relationship dissolution affects a large proportion of the population and is an important public health issue. Consequently, identifying risk factors for divorce and relationship dissolution is an important task. Knowledge about such factors may, among other things, give health workers the opportunity to target preventive interventions to those couples that are at increased risk of marital dissolution.

At first glance, there seems to exist a straight-forward impact of relationship satisfaction on dissolution. This topic has been studied extensively in the US, and there is a well-documented positive relationship between marital satisfaction and marital stability [27, 37]. One study found that it is possible to predict divorce quite accurately using models that include marital dissatisfaction measures, thoughts about divorce/separation, and certain interaction patterns of the partners [29]. There are few European studies that address relationship predictors of divorce. However, one retrospective study of divorced individuals in Germany, Italy, and Switzerland suggested that low commitment and deficits in interpersonal competencies are central predictors for divorce [11]. In summary there is a strong link between dissatisfaction and dissolution, yet this relationship may be attenuated by an assortment of factors [57].

There is ample evidence of a cross-sectional association between mental health problems, such as depression and divorce/relationship dissolution [26, 73]. Depression is associated with increased risk of divorce in both men and women [16, 40], but the association is nevertheless most likely bidirectional. Not only is depression associated with subsequent divorce, suggesting that depression (or its consequences) might impair relationships to the point of dissolution, but loss of a romantic relationship also confers significant risk of depression [7, 40, 47]. Interestingly, in some cases, depression may be associated with staying in an unhappy marriage [22]. The main picture that emerges is nevertheless that higher levels of symptoms of anxiety and depression are associated with relationship dissolution.

Researchers differentiate between acute life events and persistent strain [15], and both are explicitly defined categories of events and difficulties that are characterized by a high degree of threat and unpleasantness and by a high likelihood of prolonged consequences [50]. It can be hypothesized that acute life events, such as serious illness, have a considerable effect on marriage, and studies have investigated whether cancer patients are at increased risk of divorce. The findings have been mixed: One study found that breast cancer does not appear to be associated with marital breakdown [24], while a Danish population study found increased risk of divorce among survivors of cervical cancer [18]. Regarding enduring strain, there is evidence suggesting that economic hardship or instability in the household increases the likelihood of marriage dissolution [20, 43, 56]. One longitudinal study, measuring strain with a summative indicator (adding strain from different domains such as job, children, finances, and daily hassles) suggested a long-term association between strain and marital outcomes [10].

A number of studies have investigated the relationships between a variety of socio-demographic risk factors and marriage dissolution. Risk factors for divorce include marrying as a teenager, having no children from the current marriage, bringing children from a previous union into a marriage, being in a second or higher order marriage, cohabiting prior to marriage, having no religious affiliation, not having the same religion as one’s spouse, living in an urban area, and growing up in a household without two continuously married parents [12, 13, 62, 64, 68, 70]. Most of these predictors have remained relatively stable over the last several decades [4]. The relation between education and relationship dissolution is unclear. Investigations conducted in the US and Scandinavia have found that higher educational levels for wives [34] and for both spouses [36, 45, 54] are negatively associated with divorce risk. However, research findings suggest that the relationship between education and divorce varies among European countries [31, 46]. When the husband is unemployed, dissolution rates increase [36, 43]. Fewer studies have examined the relationships between divorce and wives’ unemployment, but results from Scandinavia have shown a divorce-promoting effect [30]. Age at marriage is perhaps the most consistent predictor of marital instability: almost all previous research has found that marriage at young age leads to an increased risk of divorce [71].

### Interaction effects

To our knowledge, only a few of the studies investigating predictors of relationship dissolution have had enough statistical power to determine interaction effects between relationship variables and other factors. The results from one study suggested interaction effects between factors such as race and relationship satisfaction [14]. Another study reported that individuals with higher levels of education are more likely than those with less education to cite incompatibility with the partner as the cause of divorce [6].

### Aims of the study

This study of a large cohort of women and their male partners addressed two research questions. The first was: what is the role of men’s and women’s risk factors in future relationship dissolution? Based on the previous research, we aimed to investigate the contribution of a set of risk factors that might be associated with relationship dissolution, such as: relationship dissatisfaction, emotional distress, enduring strain, and demographic variables (low educational level and unemployment). The outcome variable was relationship dissolution over a 39-month period. Because non-marital cohabitation is commonly accepted in Norway as an alternative to legal marriage and is well established as a childbearing institution, we combined data from cohabiting couples and married couples. We hypothesised that relationship dissatisfaction would be of particular importance for the men and women in our sample. The second research question addressed in this study was: are there interaction effects between relationship dissatisfaction and other risk factors for dissolution?

## Method

### Participants and procedures

The present study used questionnaire data from the population-based Mother and Child Cohort Study (MoBa) conducted at the Norwegian Institute of Public Health. In brief, MoBa is a cohort study of more than 100,000 pregnancies recruited from 1999 to 2008 and presents a broad basis to study health development. All hospitals and maternity units in Norway with more than 100 births annually were included during certain periods of the study [48]. The assessment points in the cohort study were the 17th (t1) and 30th (t2) gestational weeks and 6, 18, and 36 months postpartum (t3–t5). Later follow ups of the MoBa sample are ongoing [48].

Women undergoing their first routine ultrasound examination at gestation week 17–18, were invited to participate with their male partners. The women received a postal invitation to participate in the MoBa together with their appointment cards for the ultrasound scan (http://www.fhi.no/morogbarn). More than 90 % of the fathers accompanied their partners to the ultrasound examination and were then asked to take part in the study. The participation rate in MoBa was 38.5 % for women and 32.2 % for men, respectively. The response rate at 17 weeks gestation (t1) among the subjects who consented to participate was 95.3 % for women and 94.7 % for men. Only the women were followed up at later time points (t2–t5). The response rate was 92 % at t2 (gestational week 30), 86 % at t3 (6 months post partum), 74 % at t4 (18 months post partum), and 61 % at t5 (36 months postpartum).

The current study was based on the Version 4 of the quality-assured data files released for research in 2008. At that time, 90,190 of the planned sample of 100,000 women and 71,648 of their partners had been recruited and returned the questionnaire at t1. Because the pregnant women and their families were recruited sequentially over a 10-year period, only 46,188 women had been in the study long enough to be invited to participate at t5. Of these, 28,175 women had returned the fifth questionnaire (t5, 36 months postpartum) and 19,106 participants had responded to all questionnaires. The sample has been found to be slightly biased with regard to some demographic variables, but not biased in terms of associations between variables [51]. As expected because of the large number of questions included in the questionnaire, some items were not answered. Therefore, we chose to impute values for missing scores according to specific criteria (see below). After replacement of missing values, the net sample size was 18,523 couples. These couples had responses for all questionnaires (t1–t5) and complete data on all variables included in the analyses. Of these couples, 51 % were married at t1, and the vast majority of the others were co-habiting partners. When couples completed the first questionnaire, the mean age was 29.6 years (SD = 4.4) for mothers and 32.2 years (SD = 5.4) for fathers. The sample has been described in more detail elsewhere [51, 59].

### Measures

The outcome variable was coded as a dichotomous variable, whereas all independent variables were entered into the analyses as categorical variables.

#### Relationship dissolution

To measure relationship dissolution, we used one of a set of life event items: “Have you experienced divorce, separation, or relationship dissolution since returning the last questionnaire?” (yes/no) and “If yes, how painful or difficult was it for you?” (Not too bad, difficult, very difficult). The item was coded no = 0, yes = 1. In addition, we gave a positive score (yes) if they left the yes/no-question blank but had checked off how difficult the dissolution was for them. We used data from t3, t4, and t5 (filled in by the female partners) covering relationship dissolutions during the 39- month time span from gestational week 30–36 months postpartum (t2–t5).

There was also one question about current marital status on each questionnaire. This information generally corresponded well with the item described above. Still, 68 subjects reported that they were married on two succeeding questionnaires, and simultaneously reported relationship dissolution in between. These respondents were considered misclassified, and their scores were changed to 0 (no relationship dissolution).

#### Relationship dissatisfaction

To measure perceived dissatisfaction with the relationship, we used responses to the 10-item Relationship Satisfaction Scale (RS) [60] reported at gestational week 17 (t1). The scale was constructed for MoBa, and is based on typical items used in scales developed previously [9, 33]. The RS scale has shown good psychometric properties, correlates 0.92 with the Quality of Marriage Index [52], and in general shows high structural and predictive validity [60]. The scale contains 10 items, such as “I am satisfied with the relationship to my partner” and “My husband/partner and I have a close relationship”. The response categories ranged from 1 (strongly agree) to 6 (strongly disagree). The satisfaction scale was reversed to measure dissatisfaction. An index of overall relationship dissatisfaction based on the 10 items was computed as an average score across the items.

The relationship dissatisfaction scores were categorized into four groups which included approximately the lowest 25 %, the next 50 %, then again the next 15 %, and the upper 10 %. The cut-off values were 1.20, 2.00 and 2.30, respectively. The lowest category (least dissatisfied) was used as reference category. The Cronbach alpha reliability for the RS score was 0.89 for women and 0.88 for men.

#### Emotional distress

Male and female emotional distress was measured at t1 using a short version of the Hopkins Symptom Checklist (SCL-25) [78]. The SCL is a self-administered instrument designed to measure symptoms of anxiety and depression [67]. The five-item version (SCL-5) correlates 0.92 with the original version [69]. We treated the sum of the five anxiety and depression items as a global measure of mental health, hereafter termed ‘emotional distress’. The SCL-5 [69] consists of these items: “Have you been bothered by any of the following during the last 2 weeks: (1) feeling fearful; (2) nervousness or shakiness inside; (3) feeling hopeless about the future; (4) feeling blue; or (5) worrying too much about things?” The response categories were 1 = not at all, 2 = a little, 3 = quite a bit, and 4 = extremely. The Cronbach alpha reliability for the SCL-5 was 0.78 for women and 0.80 for men. The SCL-5 scores were highly skewed with a tail to the right. We recoded the emotional distress variable into three categories: (1) no reported symptoms (52.3 % of the women, 73.1 % of the men), (2) some symptoms (corresponding to a mean item score up to 1.50; 32.1 % of the women and 19.2 % of the men), (3) moderately or highly depressed (corresponding to a mean item score approximately ≥1.50 or higher; 15.6 % of the women, 7.7 % of the men). The lowest category (no reported symptoms) was chosen as reference category.

#### Socio-demographic variables

Educational level: one item that measured the educational level of the participants was included. The six response categories ranged from 9-year secondary school to >4 years at university. We reversed the scores before inclusion in the analyses, based on the hypothesis that low educational level implies an increased risk of relationship dissolution. Unemployment: one item measuring unemployment (disability retirement or out of work) was included in the analyses. The item was coded as a dichotomous variable (no = 0, yes = 1). Age: the women’s age was used as a control variable in the analyses.

#### Persistent strain and acute life events

Persistent strain during the previous year was measured at gestational week 17 (t1) for men. These items were not included in the first questionnaire completed by the women; instead, these data as well as data related to acute life events were obtained from the questionnaire completed by the women at gestational week 30 (t2). The types of life events and strain cover life events and persistent strain similar to the Life Events and Difficulties Schedule (LEDS) [15]. Persistent strain: both men and women were asked whether they had experienced any of the following three problems during the last 12 months (yes = 1, no = 0): problems at work or where you study, financial problems, or problems or conflicts with family, friends, or neighbours. In addition, the women were asked another four questions about acute life events: “Have you experienced the following during the last 12 months (yes/no): been seriously ill or injured; been involved in a serious accident, fire, or robbery; has anyone close to you been seriously ill or injured; have you lost someone close to you?” Both sexes were also asked to rate how difficult each event or strain was for them, using a three-point scale ranging from “not so difficult” to “very difficult”. A positive score (yes) was given if the yes/no-item was left blank but the follow-up question about the difficulty of the event or strain was answered. The persistent strain–variable was coded 0–3, referring to the number of problems reported during the previous 12 months. Female acute life events was coded 0–2 (the highest two categories were merged due to the low number in the highest category).

All risk factors were measured at gestational week 17 (t1), with the exception of female persistent strain and acute life events, which were measured at gestational week 30 (t2).

### Treatment of missing values

#### Replacement of missing values

Including information from participants for whom some data are missing increases the power of the analyses. We used SPSS MVA, Expectation Maximization (Graham, Hofer and MacKinnon) to impute values for missing scores on the continuously distributed scales SCL-5 and RS. The imputations were conducted separately for each scale using the remaining scale items to predict values that would best replace missing values. Imputed values were generated when respondents already had valid data for at least half of the items on the scale. In the current sample, 1.3 % of the women and 0.9 % of the men had imputed values on the SCL-5 scale, and 3.8 % of the women and 2.4 % of the men had imputed values on the RS scale.

### Ethical considerations

Informed consent was obtained from each participant, both men and women, before inclusion in the study, which was approved by the Regional Committee for Medical Research Ethics and the Norwegian Data Inspectorate. The study has been performed in accordance with the ethical standards laid down in the 1964 Declaration of Helsinki and its later amendments.

### Statistical analyses

The effects of relationship dissatisfaction, life events, emotional distress, and demographic variables on the risk of relationship dissolution were examined using logistic regression analyses. Interaction effects between relationship dissatisfaction and all the other independent variables were tested simultaneously, using logistic regression analyses. Owing to the high number of significance tests, the significance level was set to 1 % (Wald test).

To examine possible attrition bias, we checked to what extent the principal predictor variables (relationship dissatisfaction and emotional distress) predicted attrition from the sample. We used a variable indicating whether they failed to respond to at least one of the questionnaires after t1 or not (yes = 1, no = 0) as outcome variable in logistic regression analysis with relationship dissatisfaction and emotional distress (at t1) as predictors. For this purpose, the two highest categories of relationship dissatisfaction were collapsed, leaving three categories with the approximate distribution 25, 50, and 25 %. Emotional distress was categorized as in the other analyses. The lowest categories (least dissatisfied/no symptoms) were chosen as reference categories.

## Results

### Descriptive statistics

In 2009, 1.8 % of all Norwegian children were affected by the dissolution of their parents’ relationship (Statistics Norway, 2011). In 3 years, corresponding to the observational period of the current study, 5.4 % of Norwegian children will experience dissolution. In our sample, the total number of couples who experienced relationship dissolutions within the 39-month time span was 807 (4.4 % of the sample). These figures indicate that the sample is close to representative regarding dissolution rate.

The proportion of unemployment was 4.0 % for women and 2.9 % for men. 8.4 % of women and 11.0 % of men reported a low educational level (i.e. only up to 2 years of high school). The mean score on the SCL-5 (range 1–4) was 1.23 for women (SD = 0.35) and 1.12 for men (SD = 0.27). Mean scores regarding relationship dissatisfaction (reversed RS scores; range 1–6) was 1.62 for women (SD = 0.57) and 1.65 for men (SD = 0.55).

### Main effects of risk factors for relationship dissolution

Table 1 shows the results of logistic regression analysis with relationship dissatisfaction, emotional distress, acute life events, persistent strain, and demographic variables as single predictors (unadjusted/crude OR), and simultaneously included (adjusted OR).

**Table 1:** Relationship dissolution in 18,523 couples over 39 months: crude and adjusted odds-ratios with 95 % confidence intervals (CI)

Risk factor	Range	% exposed	Crude odds ratio (95 % CI)	*p* ^a^	Adjusted odds ratio (95 % CI)	*p* ^a^
Maternal age (ref = 35 years or older)		13.6		<0.01		<0.01
<20		0.7	14.99 (9.87–22.77)	<0.01	7.93 (4.99–12.63)	<0.01
20–24		10.8	2.79 (2.18–3.58)	<0.01	2.30 (1. 76–3.01)	<0.01
25–29		37.6	0.95 (0.75–1.20)	0.66	1.06 (0.83–1.35)	0.67
30–34		37.3	0.75 (0.59–0.96)	0.02	0.87 (0.68–1.13)	0.30
Female relationship dissatisfaction (ref = dissatisfaction score <1.20)	1–4	22.4		<0.01		<0.01
1.20–1.99 (somewhat)		55.0	1.58 (1.25–2.00)	<0.01	1.35 (1.05–1.74)	0.02
2.00–2.29 (moderately)		13.2	2.80 (2.14–3.67)	<0.01	2.04 (1.52–2.75)	<0.01
2.30–6.00 (most dissatisfied)		9.5	6.75 (5.26–8.68)	<0.01	3.26 (2.40–4.44)	<0.01
Male relationship dissatisfaction (ref = dissatisfaction score <1.20)	1–4	27.9		<0.01		<0.01
1.20–1.99 (somewhat)		46.3	1.29 (1.06–1.59)	0.012	1.04 (0.84–1.30)	0.72
2.00–2.29 (moderately)		16.8	2.08 (1.66–2.61)	<0.01	1.32 (1.02–1.70)	0.03
2.30–6.00 (most dissatisfied)		9.1	4.49 (3.58–5.62)	<0.01	1.72 (1.29–2.28)	<0.01
Female emotional distress (ref = no reported symptoms)	1–3	52.3		<0.01		<0.01
Some symptoms		32.1	1.82 (1.53–2.16)	<0.01	1.28 (1.07–1.54)	<0.01
Moderately or highly depressed		15.6	3.62 (3.03–4.32)	<0.01	1.51 (1.23–1.86)	<0.01
Male emotional distress (ref = no reported symptoms)	1–3	73.1		<0.01		0.014
Some symptoms		19.2	1.57 (1.33–1.87)	<0.01	1.13 (0.94–1.36)	0.21
Moderately or highly depressed		7.7	3.09 (2.54–3.76)	<0.01	1.41 (1.12–1.78)	<0.01
Female unemployment	0,1	4.0	2.23 (1.71–2.90)	<0.01	1.27 (0.95–1.69)	0.11
Male unemployment	0,1	2.9	2.69 (2.03–3.58)	<0.01	1.23 (0.89–1.69)	0.21
Low education, female (ref: >4 years at university/college)	1–6	21.8		<0.01		<0.01
4 year university degree		45.7	1.37 (1.08–1.75)	0.010	1.06 (0.82–1.37)	0.64
3 year high school general studies, junior college		12.2	2.67 (2.04–3.51)	<0.01	1.37 (1.01–1.84)	0.04
Vocational course		11.9	3.70 (2.86–4.79)	<0.01	1.51 (1.13–2.03)	<0.01
1–2 year high school		4.0	4.94 (3.61–6.77)	<0.01	1.76 (1.23–2.51)	<0.01
9 year secondary school		4.4	3.96 (2.87–5.47)	<0.01	1.55 (1.08–2.21)	0.017
Low education, male (ref: >4 years at university/college)	1–6	22.3		<0.01		<0.01
4 year university degree		30.3	1.49 (1.12–1.97)	<0.01	1.27 (0.95–1.70)	0.11
3 year high school general studies, junior college		9.9	3.12 (2.30–4.23)	<0.01	2.11 (1.53–2.93)	<0.01
Vocational course		26.4	3.28 (2.52–4.24)	<0.01	1.94 (1.46–2.58)	<0.01
1–2 year high school		5.9	5.88 (4.34–7.96)	<0.01	2.95 (2.11–4.13)	<0.01
9 year secondary school		5.1	6.09 (4.46–8.31)	<0.01	2.80 (1.99–3.95)	<0.01
Female persistent strain (ref: no reported persistent strain the previous year)	0–3	57.4		<0.01		<0.01
One reported problem		29.7	1.59 (1.35–1.88)	<0.01	1.20 (1.00–1.43)	0.046
Two reported problems		11.0	2.94 (2.42–3.56)	<0.01	1.57 (1.26–1.94)	<0.01
Three reported problems		1.9	6.32 (4.67–8.55)	<0.01	2.33 (1.65–3.30)	<0.01
Male persistent strain (ref: no reported persistent strain the previous year)	0–3	53.9		<0.01		<0.01
One reported problem		31.1	1.39 (1.18–1.65)	<0.01	1.11 (0.92–1.32)	0.274
Two reported problems		12.2	2.39 (1.97–2.90)	<0.01	1.21 (0.97–1.50)	0.093
Three reported problems		2.8	5.25 (4.01–6.87)	<0.01	1.87 (1.37–2.55)	<0.01
Female acute life events^b^ (ref: no reported acute life events the previous year)	0–2	75.1		<0.01		0.21
One reported life event		19.3	1.35 (1.14–1.60)	<0.01	1.18 (0.98–1.41)	0.079
2–3 reported life events		5.6	1.42 (1.07–1.87)	0.014	1.04 (0.77–1.40)	0.816

^a^Wald’s test

^b^The men did not answer questions about acute life events

The overall *p* values for each variable are shown in the same rows as the reference categories

As seen in Table 1, all factors had significant crude effects on the risk of relationship dissolution (*p* < 0.01). In addition to the highest level of female (OR = 6.75) and male (OR = 4.49) relationship dissatisfaction, age less than 20 years (OR = 14.99), low education for both men and women, and high level of emotional distress in both men and women were among the factors with the strongest predictive value. High level of persistent strain was also a strong predictor.

In the multivariate analysis, eight of 12 risk factors were significantly associated with relationship dissolution (*p* < 0.01) after mutually controlling for all variables. Except for young maternal age, high relationship dissatisfaction in women and low education in men were the strongest predictors of relationship dissolution. Another strong predictor was women’s persistent strain.

The effect of the women’s relationship dissatisfaction was nonlinear, such that the effect was particularly strong for the most dissatisfied group (OR = 3.26). For these (≈10 % most dissatisfied) women the risk of dissolution was more than three times higher than for the low score group (the ≈25 % most satisfied). Women moderately dissatisfied with their relationship (≈15 %) had approximately two times higher risk of dissolution (OR = 2.04). For men, relationship dissatisfaction had a clear nonlinear effect in which the risk of dissolution was significantly higher at a 1 % level (OR = 1.72) only for the high score group (upper ≈10 %).

The results showed no clear non-linear trends for female emotional distress (OR = 1.28 for the mid-category, OR = 1.51 for the moderately/highly depressed group). For men, there was a non-linear trend in which only the most depressed men had an increased risk of relationship dissolution (OR = 1.41).

For low education, the results suggested mainly linear effects. There was almost three times higher risk of dissolution for the lowest educated men, and almost two times higher risk for the lowest educated women. The results showed a steady increase in risk of dissolution with number of reported persistent strain problems. The risk almost doubled from the lowest to the highest strain category in men, and more than doubled in women.

The results did not show significant effects for female acute life events or for unemployment in any genders. Also, the overall effect of male emotional distress did not reach full significance at the 0.01 level (*p* = 0.014).

For all predictors except low educational level, the adjusted effects of female variables tended to be stronger than the adjusted effects of male variables. Regarding relationship dissatisfaction, the confidence intervals for men and women were only overlapping for some categories, suggesting that women’s dissatisfaction with the relationship represents a stronger risk of dissolution than men’s dissatisfaction. However, male and female estimates from the same analysis are not statistically independent, so formal significance testing of the sex differences is not feasible using standard analysis techniques.

### Interaction effects

Interaction effects were investigated for relationship dissatisfaction with the other variables in the analyses. We conducted simultaneous tests for all interaction terms. No significant interaction effects (all *p*-values ≥0.046) were found.

### Attrition analysis

Both relationship dissatisfaction and emotional distress predicted attrition from the sample. The ORs for relationship dissatisfaction were 0.90, *p* < 0.001 (moderately dissatisfied) and 1.06, NS (highly dissatisfied) for women and 1.28, *p* < 0.001 (moderately) and 0.96, NS (highly) for men. The corresponding values for emotional distress were 1.00, NS (moderately distressed) and 1.14, *p* < 0.001 (highly distressed) for women and 1.09*, p* < 0.001 (moderately) and 1.23, *p* < 0.001 (highly) for men.

## Discussion

### Main effects

The first aim of the current large-scale study was to investigate risk factors for relationship dissolution in 18,523 couples in Norway. Men’s and women’s relationship dissatisfaction, emotional distress, low educational level, persistent strains, and young age of the female partner were all significantly associated (*p* < 0.01) with relationship dissolution after mutually controlling for all variables. Except for young age, female relationship dissatisfaction was the strongest predictor for relationship dissolution. 22.4 % of the female sample reported relationship dissatisfaction close to the minimum score. When compared with this group, the risk of relationship dissolution, calculated from the adjusted OR, was more than three times higher for the ≈10 % most dissatisfied women. Male relationship dissatisfaction also predicted dissolution, although this association appeared to be weaker.

Our findings are in line with our hypothesis, and some results from previous studies conducted in the US that reported on an effect of relationship dissatisfaction on dissolution [27, 37]. Although few European studies have been conducted, one exception is a relatively small retrospective study of 662 divorced individuals suggesting that low commitment and deficits in interpersonal competencies are central predictors for divorce [11]. The current large-scale study adds to the knowledge in the field by demonstrating that relationship dissatisfaction (both for men and women) is an important risk factor for relationship dissolution.

The causes of relationship dissolution may differ for men and women. Certain variables, such as experiencing affirmation by one’s spouse, predict marital stability for husbands, but not for wives [55]. When asked what caused their divorce, men and women identify different variables, leading some researchers to suggest that there may be “his” and “hers” divorces [27]. The present results suggest a sex difference regarding the importance of relationship dissatisfaction: female dissatisfaction seems to increase the risk of relationship dissolution more than male dissatisfaction.

Our finding that female relationship dissatisfaction appeared to have a stronger effect than male relationship dissatisfaction contrasts to results from a national survey in the US in the 1980s and the early 1990s [27]. The US study seems to suggest that marital happiness in men predicts dissolution more strongly than does happiness in women [27]. One possible explanation for these conflicting results may be differences in socio-economic conditions in the US at that time versus present day Norway. Specifically, young Norwegian women today are highly educated compared to women in the US 30 years ago, there is high regard for equal rights between men and women in Norway, and the Norwegian economy is strong. In addition, the social system is well developed with extensive rights for single parents, for example. When compared with other times and to societies in which women were more dependent on their husbands, economically and otherwise, women in our sample may feel freer to end a relationship with which they are dissatisfied. Another explanation of the results in the US study was that unhappiness was reported for the previous year. When more proximate evaluations such as current marital trouble were considered, the wives’ evaluations seemed to be more salient.

In the present study, high levels of emotional distress, as experienced by both men and women was associated with an increased risk of relationship dissolution, in agreement with some previous research [16, 39]. Both sexes’ persistent strain was also significantly related to an increased risk of dissolution in the groups with highest scores. Previous studies have shown divergent results concerning the influence of major persistent strain on marriage [21, 63]. One reason for this may be that different studies have investigated different types of strain. The occurrence of acute life events had no significant effect on relationship dissolution in the current study. It seems reasonable that risk factors that are stable over time put more strain on individuals and on a relationship compared to acute events [61].

The findings from the current study suggest that low educational level (for both men and women) is associated with relationship dissolution. Results from previous studies are contradictory, and the relationship between education and divorce varies in different European countries. One comparative study found that education and divorce/relationship dissolution were positively associated in some countries (Greece, Italy), negatively associated in some countries (Austria, Lithuania), and not associated in some countries (Finland, Hungary, Sweden, and Switzerland) [31]. The authors concluded that education is positively associated with divorce in countries where marital dissolution is relatively uncommon and the social and economic costs are high, and there is no relationship or a negative relationship in countries where marital dissolution is relatively common and the costs are relatively low. Consistent with this notion, a national survey addressed historical developments on the effect of five social determinants of divorce in the Netherlands. The results indicated that in this country, the association between education and divorce tended to be positive in earlier marriage cohorts and negative in more recent cohorts [23]. That is, historically (when divorce was uncommon), people who were more educated were more likely to divorce than were those with less education. Currently, those who are less educated are more likely to divorce than those with more education. These findings are in accordance with the results in the current study.

### Interaction effects

This study found no significant interaction effects between relationship dissatisfaction and the other variables in the analyses. These negative findings suggest that relationship dissatisfaction does not moderate the effects of the other variables much, and the other variables do not noticeably moderate the effects of relationship dissatisfaction on dissolution. However, even with a large sample like ours the power to detect interaction effects is limited, and some interaction effects may have been left undetected.

### Strengths and limitations

The most important strengths of this study are its high statistical power due to the large number of participants and its precise estimation of main effects. Small effects and even negative results were still highly informative because of the narrow confidence intervals. The large sample also allowed us to detect interaction effects. The true prospective design is also an advantage compared to the many previous retrospective studies. There are few European studies that address relationship predictors of divorce/dissolution [4]. The current study used self-reported data from both male and female partners. By examining the role of both partners’ relationship dissatisfaction, in addition to well-known risk factors, the current study will add to the present knowledge in this field.

This study also had some limitations. First, the validity and reliability of the outcome measure might be less than optimal, which may have deflated the estimates. Second, the participation rates were 38.5 % for invited women and 32.2 % for invited men. The response rate at t5 (36 months postpartum) was 61 % for the women who had been in the study long enough to be invited to participate at this time point. However, this low response rate is not uncommon for large epidemiological studies and does not necessarily imply an unrepresentative sample [32].

The results from our attrition analysis showed only moderate selection of women who dropped out of the study regarding relationship dissatisfaction and emotional distress. Nevertheless, and despite an observed dissolution rate in our sample which does not deviate much from what is expected in the population, we cannot rule out the possibility of a more severe attrition bias in the first place, since the participation rate for entrance to the MoBa study is rather low. Previous results have shown significant mean differences in prevalence estimates between the MoBa cohort and the total population of young women for certain variables, but no statistically significant differences in exposure–outcome associations [51]. This is consistent with the notion that our sample may be unsuitable for reliable estimation of descriptive statistics, but the low response rate is not expected to affect dramatically our risk estimates [32].

Our sample consisted of couples in a certain phase of life: they were expecting a baby at t1 and had responsibility for young children at t5. About 50 % of the women already had children when the investigation was undertaken. Consequently, we do not know to what extent the results can be generalized to couples in the general population. The previous results regarding the association between having children and relationship dissolution are contradictory [8, 75, 76]. However, because the present results are mainly in line with the findings from earlier studies on couples in different phases of life, it seems that our findings can be generalized to couples in other phases of life. The fact that there was no significant interaction effect between relationship dissatisfaction and age supports this assumption. Nevertheless, because of large cultural differences, our results may not be generalized to non-Western cultures and especially not to societies in which divorce is much less accepted. Most of the previous research referred to in the current study was undertaken in Europe and the US, and to our knowledge, there have been few studies conducted in non-Western societies.

Our results are not fully informative regarding the direction of causality. Although we find it likely that emotional distress may be a risk factor for relationship dissolution, we cannot rule out the possibility of a reversed causal pathway. The real associations between a risk factor like emotional distress, relationship dissatisfaction and relationship dissolution are probably to some extent bidirectional. Besides, there may be extraneous “third” variables that can influence both relationship dissatisfaction and relationship dissolution, like stable personality characteristics. Ideally, future studies are needed that follow the subjects from before the relationship is established until after it is dissolved, but such data are of course difficult to obtain.

### Implications and conclusion

Understanding factors that impact marital stability is important, as many studies have demonstrated negative consequences of relationship dissolution for both adults and children. Our investigation adds to this body of knowledge and confirms the significance of certain risk factors, such as relationship dissatisfaction, emotional distress, persistent strain, and low educational level, in predicting relationship dissolution. This knowledge presents policy makers, health authorities, and health workers with the opportunity to better target preventive interventions for couples at increased risk of marital dissolution. Among all the risk factors in the study, female relationship dissatisfaction appeared to be the most important factor in addition to age. Compared to the situation several decades ago, when practical and economical factors seemed most important, relationship quality seems to be more important for many couples today in terms of deciding to maintain or dissolve a relationship. Making arrangements that facilitate and foster a good relationship is important both on the societal level and on the individual level. Because relationship dissolution affects a large proportion of the population, this remains an important public health issue.
